# Neuroinflammation: friend and foe for ischemic stroke

**DOI:** 10.1186/s12974-019-1516-2

**Published:** 2019-07-10

**Authors:** Richard L. Jayaraj, Sheikh Azimullah, Rami Beiram, Fakhreya Y. Jalal, Gary A. Rosenberg

**Affiliations:** 1 Department of Pharmacology and Therapeutics, College of Medicine and Health Sciences, United Arab Emirates University, Al-Ain, UAE; 2 Department of Neurology, University of New Mexico Health Sciences Center, Albuquerque, NM 87131 USA

**Keywords:** Stroke, Neuroinflammation, Ischemia, Blood-brain barrier

## Abstract

Stroke, the third leading cause of death and disability worldwide, is undergoing a change in perspective with the emergence of new ideas on neurodegeneration. The concept that stroke is a disorder solely of blood vessels has been expanded to include the effects of a detrimental interaction between glia, neurons, vascular cells, and matrix components, which is collectively referred to as the neurovascular unit. Following the acute stroke, the majority of which are ischemic, there is secondary neuroinflammation that both promotes further injury, resulting in cell death, but conversely plays a beneficial role, by promoting recovery. The proinflammatory signals from immune mediators rapidly activate resident cells and influence infiltration of a wide range of inflammatory cells (neutrophils, monocytes/macrophages, different subtypes of T cells, and other inflammatory cells) into the ischemic region exacerbating brain damage. In this review, we discuss how neuroinflammation has both beneficial as well as detrimental roles and recent therapeutic strategies to combat pathological responses. Here, we also focus on time-dependent entry of immune cells to the ischemic area and the impact of other pathological mediators, including oxidative stress, excitotoxicity, matrix metalloproteinases (MMPs), high-mobility group box 1 (HMGB1), arachidonic acid metabolites, mitogen-activated protein kinase (MAPK), and post-translational modifications that could potentially perpetuate ischemic brain damage after the acute injury. Understanding the time-dependent role of inflammatory factors could help in developing new diagnostic, prognostic, and therapeutic neuroprotective strategies for post-stroke inflammation.

## Introduction

Although stroke is the third leading cause of death after heart disease and cancer, it leads to permanent disabilities in 80% of survivors [1, 2]. Stroke can be classified into ischemic or hemorrhagic, in which 85% of strokes are ischemic [3]. Worldwide, cerebrovascular disease and in particular stroke causes a large percentage (47–67%) of disability-adjusted life years and death [4]. Ischemic stroke is characterized by arterial occlusion due to embolus or thrombus [5]. The functional and metabolic irregularities that occur during ischemic stroke largely depend on the artery that is occluded, which in turn determines the size of the ischemic area in the brain [6].

In 2014, a review by Mehndiratta et al. reported that there is an increase in incidence of ischemic stroke subtypes in Asian countries, since Arab countries have identical epidemiological characteristics to other Asian countries, that would suggest an increase in ischemic stroke in Arab countries as well [7]. Moreover, reports from a population-based screening program indicate that there is a very high risk for cardiovascular disease, especially diabetes and obesity in the United Arab Emirates [8, 9]. These reports indicate that there will be a substantial increase in stroke burden, which results in substantial economic and societal costs [10]. Thus, understanding molecular neuroinflammatory mechanisms could significantly potentiate therapeutic development.

While the changes due to loss of glucose and oxygen, including the triggering of a series of oxidative, biochemical, and hormonal reactions culminating in microvasculature injury and blood-brain barrier (BBB) disruption, are well established, less well understood are secondary inflammatory cells and their mechanisms that in turn initiate excitotoxicity, reactive oxygen species (ROS), and nitric oxidative species (NOS), and these events will be the focus of the following discussion [11].

## Impact of inflammatory cells on stroke

Various studies demonstrate that apart from neurons, other cells are involved in the pathogenesis of ischemia. Moreover, neuronal, glial, and vascular elements together form a functional “neurovascular unit.” Following ischemic stroke, microglia and astrocytes are activated within hours, leading to the production of cytokines and chemokines [6] and resulting in infiltration of leukocytes [12, 13].

### Microglia

Microglia, the resident innate immune cells of the brain that represent 5–20 % of the glial population, are activated after ischemic stroke resulting in morphological and phenotypical changes [14]. Activated microglia act similarly to macrophages during systemic inflammation, and they have the ability to phagocytose (clearing foreign organisms and cellular debris) and produce cytokines and MMPs that can compromise BBB function [13]. Whereas, quiescent resting microglia are in a ramified state defined by a small cell soma with wide branches projecting out [15, 16]. However, following ischemia, microglia cells are activated but the precise mechanisms are still not clear. Transient focal cerebral ischemia caused by 15 min of transient middle cerebral artery occlusion (tMCAO) in spontaneously hypertensive stroke-prone rat (SHRSP) leads to activation of microglia in the cerebral cortex of the ischemic hemisphere, and the severity of injury may be reflected in the state of microglial activation [17]. Microglia-mediated neurotoxicity is augmented by the production of ROS via NADPH oxidase [18], cytokines [6], and MMPs [13, 19]. Sun et al. demonstrated that inhibition of microglial activation by 2% isoflurane in transient focal cerebral ischemia rats (reperfusion after 90 min of ischemia) reduced infarct volume, attenuated apoptosis, and significantly decreased microglial activation in ischemic penumbra [20]. Microglial activation also causes secondary death in the penumbra region [21]. Various studies have reported that CD14 receptors (activated by iNOS) followed by toll-like receptor 4 (TLR4) are expressed in activated microglia in infarct brain region, and hence, this could be a possible microglial activation mechanism [22, 23]. In 2017, McDonough et al. demonstrated that sequential exposure of wild-type and TLR4^−/−^ microglia to hypoxia/hyperglycemia (H/H) and normoxia/normoglycemia (N/N) resulted in enhanced expression of type-1 interferon-stimulated genes (ISG) in H/H-N/N wild-type microglia and not in TLR4^−/−^ microglia. Type-1 interferons such as interferon-α and interferon-β activate interferon-α/β receptor (IFNAR) complex and microglial type-1 interferon-stimulated gene expression and were dependent on IFNAR1. During H/H conditions, interferon-β induces secretion of ISG chemokines. Enhanced expression of ISG in microglia and microglial ISG chemokine response to TLR4 agonists were dependent on IFNAR1 and TLR4. To conclude, interferon triggered gene expression in microglia plays a major role in ischemia/reperfusion injury [24]. However, during ischemic stroke, activated microglia have been shown to play a dual role, and they secrete pro-inflammatory cytokines resulting in further damage [6] and also secrete anti-inflammatory factors [25, 26]. Three and 7 days after MCAO, edaravone, a free radical scavenger that mimics glutathione peroxidase (GPx), diminishes microglial activation and early accumulation of oxidative products in rats [27]. Similarly, multiple exposures to hyperbaric oxygen (HBO) reduced infarct volume by decreasing microglial activation [28]. Surprisingly, impaired microglial activation markedly increased infarct size and number of apoptotic neurons following ischemia (MCAO, 1 h followed by 24- or 72-h reperfusion periods) supporting the dual role of microglia [29]. Following ischemia, activated macrophages can be detected as soon as 2 h. Between 22–46 h, both blood born and resident macrophages are dispersed over the entire lesion and stay detectable for up to 1 week in mice following the 30-min ischemic insult [30]. Microglia found around the ischemic tissue migrate towards the ischemic lesion and remain in close association with neurons for a process called “capping” (following neuron death, capping helps in early recognition and quick phagocytic removal of dead neurons) [31, 32]. Genetic deficiency of lymphocyte function-associated antigen 1 (LFA-1) abolishes the ability of microglia to migrate towards injured neurons [33]. Also, microglia play a major role in the production of growth factor TGF-β1, which represents a neuroprotective property [34]. Recently, Jin et al. demonstrated that depletion of microglia by dual colony-stimulating factor-1 inhibitor, PLX3397 intensifies brain infarction and neurodeficits. At day 1 and 3 after 60-min transient MCAO and reperfusion, microglia depletion also exacerbates leukocyte infiltration, expression of inflammatory mediators, and neuronal death in mice. This pathological mechanism is not only solely dependent on lymphocytes and monocytes but also due to astrocyte-mediated inflammatory factors. Hence, the presence of microglia prevented the secretion of astrocyte-mediated inflammatory factors during ischemia [35]. Also, supporting this, microglia produce various neurotrophic factors which promote neurogenesis and plasticity [36]. Hence, following ischemia, different subsets of microglia have different roles.

### Astrocytes

Similar to microglia, astrocytes are housekeeping cells mandatory for maintenance of the central nervous system. They are actively involved in controlling ion and water homeostasis, releasing of neurotrophic factors, and scavenging transmitters released during synaptic activity, shuttle metabolite, and waste products and are involved in BBB formation [37]. Under normal physiological conditions, astrocytes take up excessive glutamate from extracellular space and convert it to glutamine for neuronal reuse, but during brain injury, the extent of damage to astrocyte might affect the ability of its glutamate uptake [37]. However, it is not clear on how ischemia exactly affects astrocytic glutamate uptake but it has been reported that the expression of glutamate transporter EAAT2 is defective during ischemia [38, 39]. Following ischemia, cytokines from neurons and glial cells lead to astrocyte reactivity hyperplasia. Astrocyte proliferation results in expression of inflammatory factors such as monocyte chemotactic protein-1, IL-1β [40], glial fibrillary acidic protein (GFAP), vimentin, and nestin that can lead to reactive gliosis and scar formation [41]. After stroke, due to failure of Na^+^, K^+^ pump, astrocytes swell, which leads to high intracerebral pressure and less cerebral perfusion [42]. Reactive astrocytes release matrix metalloproteinase 2 that causes degradation of matrix protein [43]. Reactive astrocytes also cause inhibitory condition by inducing ephrin-A5 at the lesion center disturbing axonal sprouting [44]. Following photothrombotic ischemic insult in rats, extensive astroglial response is initiated in the lesions’ core from 4 h to 1 day and reaches maximum at 4 days and persists until 28 days after onset [45]. Three days after transient global ischemia (10 min followed by reperfusion), there is a significant upregulation in the expression of iNOS, NADPH diaphorase, and GFAP in hippocampal astrocytes [46]. Post-mortem brain tissues from ischemic patients who died 3–7 days after stroke revealed an enhanced expression of IL-15 in astrocytes. Alternatively, knockdown of IL-15 in astrocytes diminished ischemic brain injury in mice subjected to transient MCAO for 60 min [47]. GFAP promoter-controlled IL-15 expressing transgenic mice showed enlarged brain infarcts, exacerbated neuronal deficits following rose bengal induced cerebral cortical photothrombotic ischemia. Additionally, GFAP/Vimentin double knockout mice showed enhanced cortical cerebral blood flow reduction and larger lesions after focal ischemia [48]. Thus, protection of non-reactive astrocytes provides significant nutrient and physical support for the survival of neurons. Calcineurin inhibitor FK506 and immunosuppressive drugs have been reported to prevent glutamate-mediated astrocyte death [49]. In addition, NF-ĸB inhibition in astrocytes protects neurons against 60-min ischemic injury in mice [50]. Astrocytes release brain-derived neurotrophic factor (BDNF), fibroblast growth factor-2, and nerve growth factor (NGF) that play an important role in neuroprotection [48, 51]. In addition to neurotrophic support, structurally, astrocytes through their endfeet possess strong association with brain capillary endothelial cells and pericytes that form BBB. During ischemia, MMP-9 disrupts the connection between astrocyte endfeet and endothelial cells by degrading basal lamina [52]. Hence, ruptured BBB acts as a major gateway for the invasion of peripheral inflammatory cells.

### Endothelial cells and blood-brain barrier

**Fig. 1:**
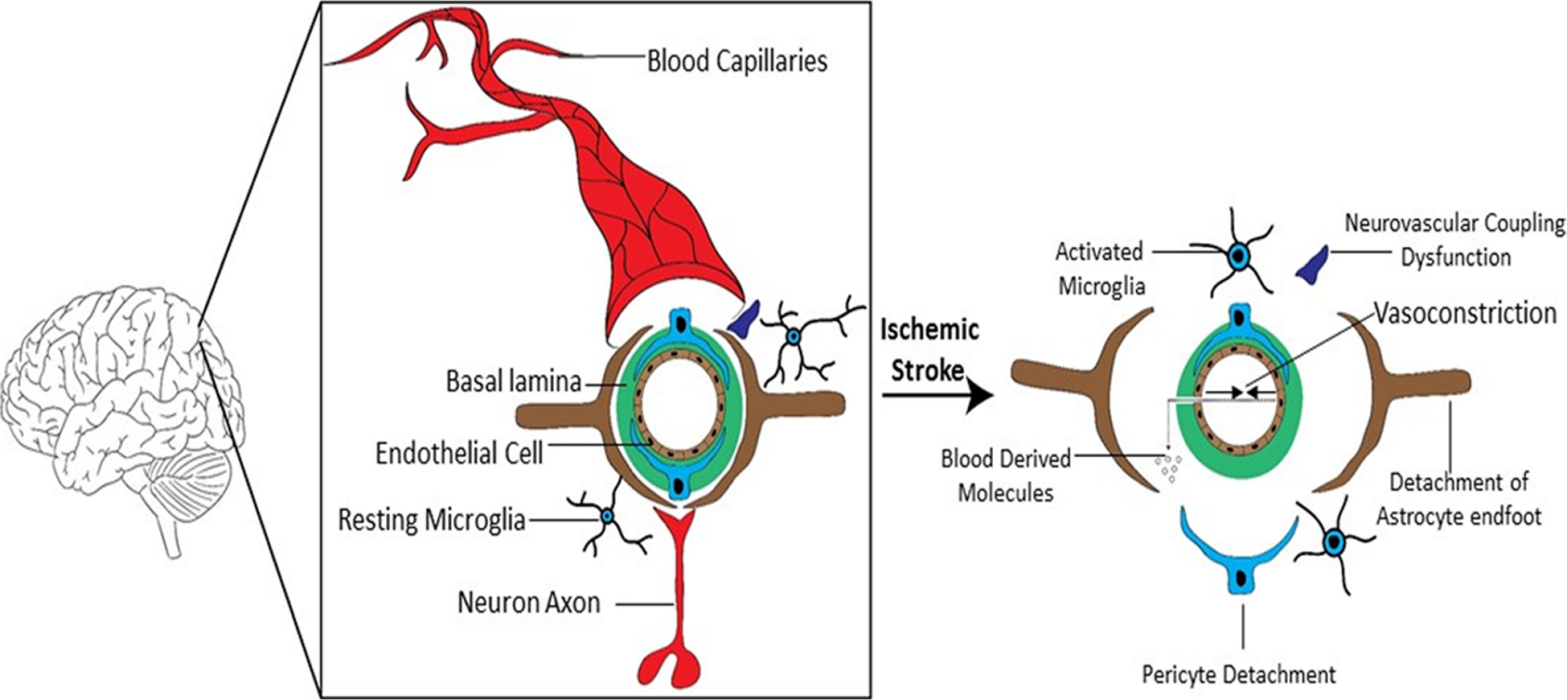
Schematic representation of the detrimental events following ischemic stroke. Stroke potentiates a cascade of ischemic events that leads to impairment of NVU resulting in BBB damage. Neurovascular unit is made of highly specialized and polarized endothelial cells interconnected by tight junction proteins that seal the brain capillaries. Astrocytes, microglia, and pericytes provide structural and functional support to the BBB. Ischemic stroke leads to NVU remodeling due to detachment of astrocytic endfoot, pericyte detachment, vasconstriction, dysfunction of neurovascular coupling, activation of microglia, and blood vessel rupture

Endothelial cells (EC) are one of the components of the neurovascular unit. They have tight junction proteins that are the major interface with the blood. The ECs are surrounded by basal lamina, which is made up of laminin, type IV collagen, fibronectin, heparin sulfate, and other extracellular molecules (Fig. 1). Pericytes are macrophage-like cells with smooth muscle properties that are contiguous with basal lamina and are involved in both angiogenesis and in injury [53]. Pericytes also play a major role in controlling neurovascular unit physiology by regulating microvascular stability, tight junction proteins, and microvessel diameter. In addition to its role in protecting the neuronal microenvironment by preventing entry into brain of potentially toxic activated plasma proteases, the BBB has specific transport systems that supply essential nutrients [54]. Following ischemic stroke, pericytes respond quickly that may either be protective or detrimental, such as detachment, constriction, and migration from microvessel walls and cell death [55]. One hour after MCAO, pericytes migrate from the basement membrane which leads to BBB permeability [56]. In addition, proinflammatory factors enhanced the expression of chemokines, interleukins, and cellular adhesion molecules in pericyte-fibroblast co-cultures [57, 58]. On the contrary, 7 days after ischemic stroke in mice, pericytes were reported to accumulate in the peri-infarct area which might support vascular repair, and additionally, they are able to differentiate into neural and vascular lineage cell [59]. Surprisingly, pericytes isolated from human stroke brain tissue are found to highly express microglial markers that may serve as stem cells generating microglia for phagocytic activity [60, 61]. Although pericytes play a major regulatory role, endothelial cells and their tight junctions provide the ultimate barrier. The endothelial cells are interconnected via clefts made up of tight junction proteins (TJP), namely occludins and claudins, which act as a major barrier between interstitial fluid and blood. Following ischemia, dynamic changes in BBB permeability results in endothelial swelling, astrocyte detachment, pericyte detachment, pericyte contraction, microglial activation, vasoconstriction, and blood vessel rupture (Fig. 1). Subtle changes in BBB permeability result in endothelial hyperpermeability to macromolecules in the peri-infarct area [62]. A biphasic pattern occurs during transient BBB disruption where on initial opening occurs 2–3 h following onset and after reperfusion (24–48 h), a second opening occurs resulting in increased intracranial pressure and vasogenic edema in mice [63, 64]. This leads to the production of adhesion molecules and proinflammatory cytokines, which, in turn, aggravates the process [65]. Moreover, 24–48 h following 2 h of MCAO with reperfusion in rats, MMPs activate microglia, macrophage, and neutrophils which secrete additional MMPs further compromising cerebral vessels [53]. However, therapeutic approaches, such as agents that induce hyperpermeability could help enhance BBB repair. Apart from dexamethasone clinical trial that failed, administration of bortezomib (proteasome inhibitor) along with dexamethasone reduced BBB permeability [66]. Novel therapeutic approaches to enhance BBB repair after stroke have been extensively reviewed elsewhere [67].

### Leukocytes

Similar to other inflammatory cells, leukocytes release proinflammatory factors in the ischemic region of the brain. Leukocytosis has been shown to be a marker of inflammatory response after stroke. Neutrophils are the first blood-borne immune cells to invade the ischemic tissue followed by monocytes [65]. After stroke, neutrophils undergo conformational changes due to extensive adhesion molecules and migrate through the endothelial vessel wall. Later, neutrophils are attracted towards the ischemic area by chemokines via concentration gradients, and neutrophils cause secondary injury by releasing proinflammatory factors, ROS, proteases, and MMPs [68]. These toxic factors impair endothelial cell membrane and basal lamina leading to BBB permeability and post-ischemia edema. Post-invasion (4–6 h) of neutrophils, monocytes adhere to the vessel walls and move towards ischemic regions with its maximum activity at 3–7 days after the insult [69]. Neutrophils play a major pathological role in acute ischemic injury and may also cause atherosclerosis and thrombus formation [70, 71]. Five hours following reperfusion, neutrophils enter the damaged area [72, 73]. In a permanent MCAO model, 12 h after the insult, peak neutrophils are reported [74]. Leukocytes potentiate ischemic injury in many ways. First, leukocytes adhere to the endothelium, which blocks the flow of erythrocytes via microvasculature, leading to cerebral no-reflow phenomenon. Second, at the surface of endothelium, activated leukocytes produce proteases, MMPs, and ROS that can significantly damage blood vessels and brain tissues. Further, biologically active substances like eicosanoids, leukotrienes, prostaglandins, and platelet-activating factor are produced when leukocytes are activated by phospholipases, leading to vasoconstriction and platelet aggregation. Finally, leukocytes (infiltrated) further exaggerate neuronal injury by activating proinflammatory factors in and around the penumbra and the infarct core [16]. Furlan et al. demonstrated that leukocytosis is significantly associated with a high degree of disability, impairment, greater risk of total anterior circulation stroke, and higher mortality [75]. Moreover, hypoxia-related genes are upregulated in leukocytes isolated from stroke patients [76]. To conclude, all these inflammatory cells play a significant role in both initiating and aggravating pathological response in stroke as well as in maintaining cellular brain homeostasis (Table 1).

**Table 1:** Beneficial and detrimental role of inflammatory cells in ischemic stroke

Cell type	Detrimental effects	Beneficial effects
Microglia/macrophages	Production of proinflammatory cytokines, including TNF and IL-1, reactive oxygen and nitrogen species, and proteases, such as MMPs. Brain microglia/macrophage phagocytose viable and functional neurons causing brain atrophy.	Resolution of inflammation (IL-10 and TGF-β release, production of arginase, and phagocytic activity). Late reparative processes by producing growth factors (IGF-1, brain-derived neurotrophic factor, and glial cell line-derived neurotrophic factor), production of neurotrophic factors, facilitation of neurogenesis and plasticity, and scavenge and removal necrotic debris
Astrocytes	Production of inflammatory mediators (e.g., TNF-α, IL-1, and MMPs). Edema formation, inhibition of axon regeneration and BBB disruption, glial scar formation, and glutamate release	Extracellular glutamate uptake, synthesis, and release of neurotrophic factors. Glial scar formation, BBB rebuilding, and neurovascular remodeling.
Neutrophils	Microvessel obstruction, ROS production, and release of MMPs that contribute to BBB damage and exacerbate inflammation, stimulation of lipid peroxidation, release of proteolytic enzymes, damage of endothelial cell membrane, increase of BBB permeability, post-ischemic edema, no-reflow phenomenon	N2 phenotype: promote resolution of inflammation
Dendritic cells	Up-regulation of MHC-II and co-stimulatory molecules that prompt the activation of lymphocytes	
T Lymphocytes	Facilitate adhesion of platelets and leukocytes to the vascular endothelium causing thromboinflammation and promoting proinflammatory pathways	Interaction of T cells with platelets may also have hemostatic effects preventing hemorrhagic transformation after severe ischemic stroke

**Fig. 2:**
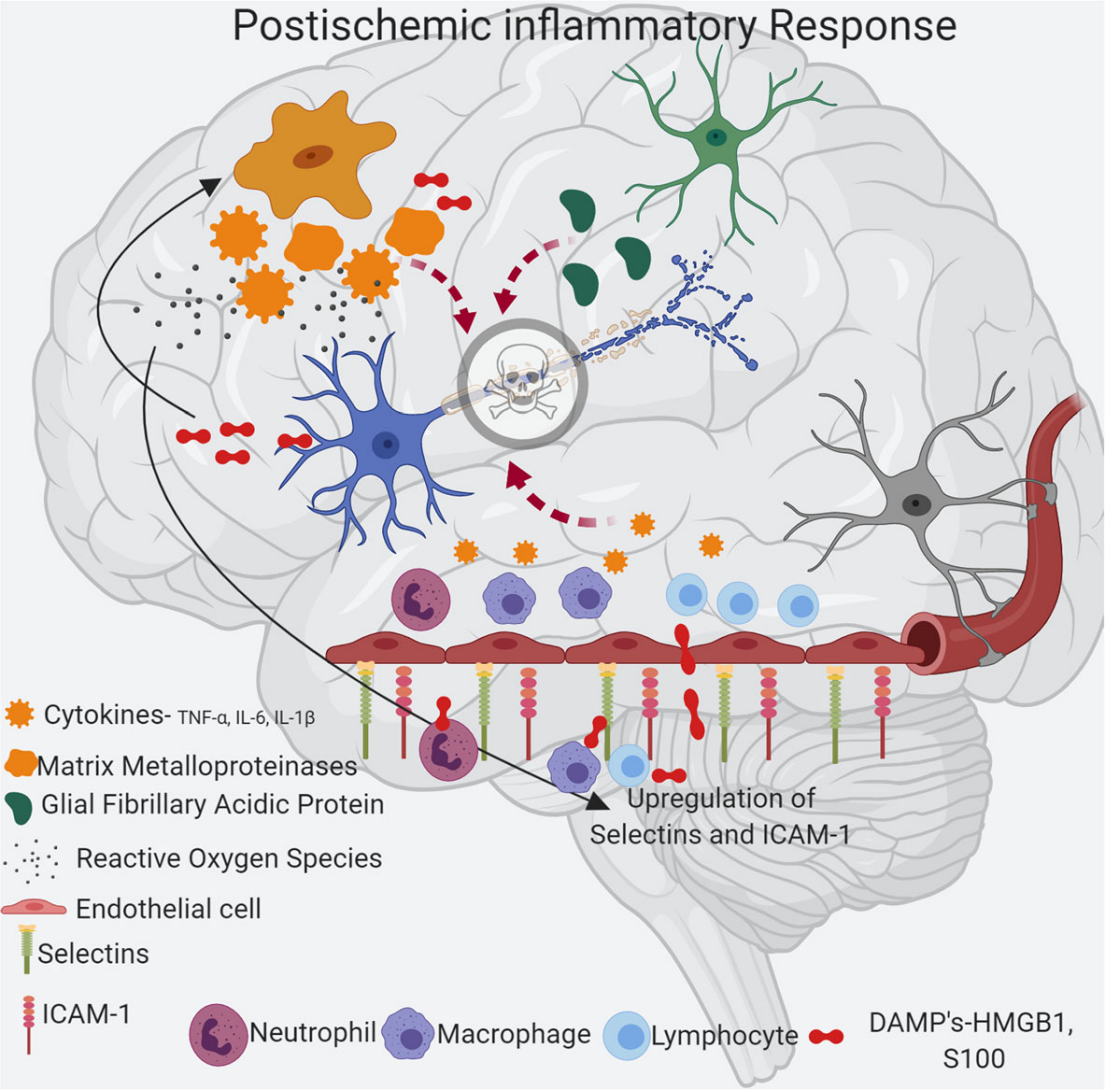
Schematic representation of post-ischemic inflammatory response in stroke. Initial ischemic event leads to oxidative stress and excitotoxicity which causes activation of microglia and astrocyte resulting in secretion of cytokines, MMP, and GFAP. These proinflammatory factors leads to upregulation of cell adhesion molecules such as ICAM-1 and selectins on endothelial cells causing inflow of blood derived inflammatory cells such as neutrophils, macrophages, and lymphocytes to the ischemic area. In addition, danger-associated molecular patterns (DAMPs) are released by dying neurons that in turn activates microglia and peripheral immune cells (neutrophil, macrophage, and lymphocyte) resulting in production of proinflammatory factors causing further activation of microglia and astrocyte. These pathological events lead to neuronal death and further increase damage to the ischemic brain

Schematic representation of the role of inflammatory cells in post-ischemic inflammation is shown in Fig. 2. However, time-dependent pathomechanisms play a significant role in determining the severity of stroke outcome.

## Time-dependent entry of immune cells during ischemic stroke

Various animal models [77–80] and clinical studies [81, 82] have demonstrated that following ischemia, apart from activation of microglial cells in the ischemic brain, infiltration of circulating cells, such as granulocytes, neutrophils, monocytes/macrophages, and T cells, occurs aggravating cell death. During an acute phase, lasting minutes to hours, the injured tissue releases reactive oxygen species (ROS) and proinflammatory factors, such as chemokines and cytokines, which induce the expression of adhesion molecules on leukocytes and on cerebral endothelial cells that in turn promote adherence and transendothelial transfer of leukocytes [83].

Following the acute phase, in the sub-acute phase, the infiltrated leukocytes further release cytokines, chemokines, and more importantly excess ROS that promotes production of matrix metalloproteinases (MMPs) especially MMP-9. Jalal et al. and various groups have proven that MMP activation intensifies an inflammatory response, leading to BBB disruption, brain edema, neuronal death, and hemorrhagic transformation due to excessive activation of proteases and resident immune cells, and further intensifies leukocyte infiltration [84, 85]. Because of the complex nature of the interaction of the proinflammatory factors with the tissues, they act in multiple roles. For example, MMP-9 not only acts as a proinflammatory factor in early ischemic brain damage, but also plays a major role in brain regeneration and neurovascular remodeling during repair [53, 86]. Hence, it is critical to understand the time course of inflammatory events and cells involved that lead to ischemic brain injury.

### Resident microglia and blood-derived macrophages

Various reports have shown that microglia, the resident macrophages of the brain, are activated within minutes after ischemic onset leading to production of various proinflammatory factors, such as interleukin-1β (IL-1β) and tumor necrosis factor-alpha (TNF-α), aggravating brain damage [14, 87]. Apart from being a proinflammatory stimulator, microglia cells also promote excitotoxic injury and ischemia in the brain [88]. Following ischemia, microglia proliferation peaks at 48–72 h and lasts for a few weeks after the initial injury [89], but contradictory to this, blood-derived leukocytes are recruited hours to a few days after initial injury [70, 90].

Since blood-derived monocytes/macrophages and reactive microglia are almost morphologically and functionally similar, it has been challenging to differentiate these cells due to lack of cell-specific markers [79, 80, 91]. Blood-derived macrophages patrol the brain to capture potential pathogens, and just like microglia, blood-derived macrophages are able to acquire a ramified morphology. Furthermore, microglia have the ability to develop a phagocytic phenotype very similar to macrophages. Hence, it is very challenging to distinguish between these two cell types, but an excellent work by Tanaka et al. using chimera mice with enhanced green fluorescent protein (GFP) bone marrow provided a useful tool to differentiate the role of resident as well as blood-derived macrophages in ischemic brain injury [80]. Many literature reports have shown that 3–4 h after stroke, blood-derived macrophages are recruited into the ischemic brain tissue [92, 93]. Schilling et al. using a transient middle cerebral artery occlusion (MCAO) chimera mouse model demonstrated that resident microglia activation predates and dominates blood-derived macrophages [91, 92]. These reports proved that following brain damage, neutrophils are the first blood-derived leukocytes observed at day 1 while GFP positive blood-derived macrophages were rarely seen at day 2 but their number peaked at day 7 and declined thenceforth. Contrary to blood-derived macrophages, even at day 1, GFP-negative microglial (resident) cells are activated rapidly, and interestingly, even at days 4 and 7, most macrophages are resident microglia (GFP-negative) in mouse models of transient and permanent MCAO [80, 94]. Recently, in an effort to understand the gene expression patterns of resident microglia and blood-derived macrophages after stroke, Kronenberg et al. used bone marrow chimerism and dual reporter transgenic fate mapping to distinguish the responses in MCAO mouse model. Biased gene expression analysis in 7 days post-stroke tissue provided 970 transcripts predominantly overexpressed in microglia and 472 transcripts expressed in blood-derived macrophages. These expression levels were further compared with transcriptomes of astrocytes, oligodendrocytes, and neuronal populations that resulted in specific genes expression in microglia and blood-derived macrophages. Genes upregulated in blood-derived macrophages are functionally involved in migration, proliferation, and calcium signaling which was further confirmed by whole-cell patch clamp technique. In addition, blood-derived macrophages were more altered towards neuroprotective M2 phenotype. Further, using Selplg^−/−^ (gene encoded for P-selectin glycoprotein ligand-1, an important factor for leukocyte recruitment in inflamed site) chimera mice, the group found that the lack of Selplg gene lowered engraftment of blood-borne cells in the ischemic brain and enhanced lesions at 7 days with poor sensorimotor performance [95]. Together, these data represent that during the first few days following cerebral ischemic injury, the majority of macrophage-like cells represent activated microglia.

### Neutrophils

Apart from microglia and blood-derived macrophages, neutrophils are one of the most important leukocytes that infiltrate the ischemic brain from 30 min to a few hours, peaking between days 1–3 following which they steadily decline over time [69]. Weston et al. reported that infiltrating neutrophils remain more than 32 days in endothelin-1-induced ischemic brain regions but their presence is masked after 3 days by the concentration of activated microglia/macrophages in the inflammatory region. Further, neutrophil infiltration elevates at day 1, spikes at day 3, and begins to decline, but is present through day 7 and 15 after cerebral ischemia. Neutrophils express various endothelial adhesion molecules within 15 min post-ischemia [96]. Six to 8 h after stroke, neutrophils have already surrounded cerebral vessels and initiates infiltration [97, 98]. Alternatively, flow cytometry analysis by Gelderblom et al. using a transient MCAO model demonstrated that other inflammatory cells such as macrophages, lymphocytes, and dendritic cells precede neutrophil influx in the ischemic hemisphere [99]. Consistent with other reports [69, 100], Gelderblom et al. demonstrated that neutrophils are the predominant cells present in the ischemic hemisphere at 3 days after MCAO [99]. Neutrophil increase has been positively correlated with infarct volume and functional deficits [69]. Neutrophil rise might be due to enhanced release from the bone marrow and spleen along with less neutrophil apoptosis [101]. In contrast to neutrophils, lymphocytes number decrease in ischemic stroke and hence neutrophil-lymphocyte ratio is increased. This ratio is closely associated with infarct size and mortality [102].

### T lymphocytes

Unlike neutrophils, T lymphocytes are recruited in the later stages of ischemic brain injury [103]. Jander et al. analyzed leukocyte infiltration in photochemically induced focal ischemia of rat parietal cortex by immunocytochemistry and reported that by day 3, numerous T cells infiltrated the border zone around the lesion by sparing the center and their number elevated between days 3 and 7 [104]. Similar to this study, Stevens et al. using a transient MCAO mouse model demonstrated that T cell (CD3^+^) infiltration increases at days 3–4 post-ischemia while activated microglia (CD11b^+^)/macrophages and neutrophils (Ly6G^+^) increased at earlier time points post-ischemia. Nevertheless, few reports demonstrated that T cells accumulate within the first 24 h in the ischemic brain following cerebral ischemia (MCAO), which might play a major role in influencing tissue inflammation and injury prior to the appearance of these cells in the extravascular space [105, 106]. Research efforts are increasing to reveal the role of different T cell subtype in ischemic stroke. Various studies have demonstrated that after stroke, CD4^+^, non-Treg T cells, CD8^+^ T cells, Tregs, and γδT cells infiltrate brain parenchyma [107]. Numerous research groups are focusing on modulating the activity of Treg cells due to their neuroprotective activity. Immuno-depletion of Treg using CD-25 specific antibody aggravated tissue loss and impaired neurological function at day 7 after MCAO-induced ischemia in mice [108]. However, different approaches to suppress Treg led to controversial results [109, 110]. Pre-clinical and clinical studies suggest that enhancing the function or number of Treg could be neuroprotective against ischemic injury [107]. However, Treg cell therapy has its own challenges such as isolation and purification of Treg, period for ex vivo Treg expansion, and anergic property of Treg.

Post-stroke pathological mechanisms aggravate cell injury due to primary cellular events, which initiate a pathological viscous cycle of inflammatory mediators that further exacerbates neuronal death. The mediators involved, their beneficial and detrimental role, and recent therapeutic strategies to combat these pathomechanisms are discussed in detail below.

## Key pathological events and pathways fueling neuroinflammation

### Oxidative stress

Oxidative stress, a disturbance in the balance between the production of reactive oxygen species (free radicals) and antioxidant defenses, is induced in cerebral ischemia especially through inflammation and reperfusion, which increases the production of ROS [111]. Xanthine oxidase and NADPH oxidase are the two key oxidative enzymes that play a major role in the production of superoxide anion, a key radical after stroke. Van Hemelrijck et al. using a rat model demonstrated that hydroxyl radicals (OH•) are elevated 2 h after stroke onset [112]. Nitric oxide (NO), a key radical, is produced by enzymatic conversion of L-arginine by three types of nitric oxide synthases (NOS) namely neuronal (nNOS), endothelial (eNOS), and inducible NOS (iNOS), which are elevated after brain ischemia. Although endothelial NOS is upregulated directly after brain ischemia, neuronal NOS and inducible NOS are upregulated only after a day and even later, respectively [112]. Nitric oxide has both beneficial and detrimental roles in cerebral ischemia. Although NO plays a major role in restoring blood supply to the ischemic area, reducing brain damage, it also reacts either with superoxide anion to form radicals or with free electrons to form peroxynitrite, contributing to lipid peroxidation, cellular toxicity, and eventually cell death [113]. In addition to the action of NO on free radicals, it also enhances the expression of adhesion molecules and inflammatory mediators, inhibits enzymes necessary for DNA replication, and promotes iron loss from cells [114]. The pathological effects of NO on brain tissue damage largely depend on the sensitivity of the cell to NO, concentration of NO, and whether the inflammatory phase is acute or chronic [115]. Knockout animal studies showed that mice lacking nNOS had reduced infarct volume, which demonstrates that NO produced by nNOS leads to tissue damage. Alternatively, injury after ischemic stroke increased in mice lacking eNOS showing the protective function of eNOS by dilating the blood vessels resulting in normal blood flow to the penumbra [116]. Furthermore, disruption of iNOS did not affect infarct volume after day 1, but after 72 h, iNOS disruption increased infarct size [117]. Various antioxidants such as vitamins, lipoic acid (LA), and *N*-acetylcysteine have been tested for efficacy in stroke [118]. Vitamin E and its analog MDL 74,722 have been reported to reduce lesion volume and behavioral impairments in rodent ischemic stroke models [119–121]. Alternatively, a follow-up study in human subjects demonstrated that vitamin E and C did not reduce the risk of ischemic stroke and did not enhance functional recovery in stroke patients, respectively [122–124]. In addition, other antioxidants such as EPC-K1, a phosphate diester of vitamin C, reduced lesion size, lipid peroxidation, and renal reperfusion injury in rat model [125, 126]. Apart from dietary antioxidants as supplements which had little or no effect in clinical trials, NXY-059, tirilazad, edaravone, and citicoline are being studied in clinical patients for its efficacy in treatment of ischemic stroke [118]. However, NXY-059 and tirilazad failed to provide clinical improvement in larger clinical trials [127, 128]. In 2001, edaravone was approved in japan to treat acute phase cerebral infarction. In 2015, edaravone was approved by Japan and in 2017, by the US Food and Drug Administration, for treatment of amyotropic lateral sclerosis [129]. However, a clinical study demonstrated that early-stage edaravone treatment delayed progression of infarction and edema and reduced acute-phase mortality, but edaravone alone did not cause any significant functional recovery [130]. Citicoline is now being reported as a potential therapeutic candidate for ischemic stroke. Citicoline, which is also called as cytidine 5’-diphosphocholine (CDP-Choline), is a combination of two molecules, cytidine, and choline. These molecules are efficient in crossing BBB and then they combine to form CDP-Choline in brain cells [68]. During ischemia, phosphatidylcholine is broken down into free radicals and fatty acids that worsen ischemic injury [131]. The hypothesized mechanism is that citicoline undergoes hydrolysis followed by dephosphorylation to form cytidine and choline and these two molecules act as substrates to re-form citicoline in the brain. This process minimize phospholipid breakdown and enhance phospholipid resynthesis for membrane repair [132]. In addition, citicoline also scavenges free radicals providing antioxidant and anti-inflammatory roles after ischemic damage [133]. Clark and his colleagues reported that 24 h post-MCAO, administration of citicoline for 28 days enhanced motor and functional recovery [131]. Due to promising biological properties, citicoline has also been administered (500 and 2000 mg, i.v.) in a randomized controlled trial [134]. However, other randomized, placebo, controlled clinical studies have reported that citicoline has limited benefits [135–137]. The controversial results might be due to methodological limitations such as odda ratios rather than risk ratio and the use of cumulative meta-analysis rather than trial sequential analysis. The results of International Citicoline Trial on Acute Stroke (ICTUS) was published by Davalos et al. where 2298 patients with moderate to severe stroke (within 24 h) of the anterior territory were randomly assigned to double-blinded treatment with 2000 mg citicoline daily or placebo for 6 weeks. Assessment for baseline characteristics and a known risk factor for stroke showed that citicoline-treated patients had well-balanced baseline characteristics risk factors for stroke. However, no significant difference was noted in primary outcome of recovery assessed by mRS score. Evaluating other pre-specified subgroups showed beneficial effects of citicoline in patients older than 70 years and in patients with less-to-moderate stroke. Thus, up-to-date meta-analysis of all clinical trials of citicoline resulted in overall beneficial effect with an odds ratio of 1.14 of achieving good clinical outcome compared to controls [138]. Despite the pros and cons, citicoline is the only promising drug in confirmative clinical trials and no other neuroprotective compound had any positive effect on subgroup analysis [138].

### Cytokines

Cytokines are immunomodulating agents and they play a major role in cell activation, proliferation, and differentiation [139]. Cytokines are generally small pleiotropic polypeptides (8–26 kDa) barely detectable in the brain with their receptors constitutively expressed at very low levels. Cytokines play a major role in upregulating the expression of cell adhesion molecules (CAM) [140, 141]. Especially, intracellular adhesion molecule 1 (ICAM 1) upregulation in the ischemic core leads to BBB disruption by aiding recruitment of leukocytes, which in turn release cytokines. BBB disruption causes migration of various inflammatory cells such as macrophages, natural killer cells, T lymphocytes, and polymorphonuclear leukocytes to the ischemic site. Various studies have demonstrated that infiltrating leukocytes and microglia elevate cytokines and some studies have reported that resident neurons and glia also produce cytokines following brain ischemia [106, 142, 143]. However, upon brain injury, the expression of pro- and anti-inflammatory cytokines is upregulated, but their spatial and temporal upregulation largely depends on the type of ischemic model used [72, 144, 145]. The three major proinflammatory cytokines are interleukin 1β (IL-1β), tumor necrosis factor-alpha (TNF-α), and interleukin-6 (IL-6) that provoke and aggravate an inflammatory response after stroke [146, 147]. Post-mortem studies demonstrate that TNF-α positive cells are observed in brains of severe ischemic stroke patients from 3 days post-stroke, staying positive until 15 months [148]. Tumor necrosis factor-α serum levels are increased within 6 h post-stroke and their levels are maintained for 10 days [149]. Similarly, IL-1β levels are elevated in the CSF with peak levels at days 2 and 3 post-stroke [150, 151]. However, some studies have shown no increase in IL-1β levels in serum and plasma, which might be due to IL-1β localization at the inflammatory site [152]. Interleukin-1β mediates ischemic, traumatic, and excitotoxic brain injury through its action on neurons, glia, and vasculature. Similar to TNF-α and IL-1β, Il-6 levels are elevated in CSF of severe stroke patients. Few studies report that CSF IL-6 levels increase within 24 h and peak at days 2 and 4 [153], while some studies report peaking at days 3 and 7 [154, 155]. However, their levels appear to be dependent on stroke type and severity. Interleukin 1β, an important mediator of neuroinflammation, upregulates the expression of IL-6. Hence, IL-1β receptor antagonist, anakinra administration, demonstrated good clinical improvement and decreased peripheral neutrophil count and IL-6 levels [156]. Alternatively, transforming growth factor-β (TGF-β) and IL-10 are anti-inflammatory cytokines that inhibit the expression of proinflammatory cytokines thereby reducing inflammation after ischemic stroke [157, 158]. These pro- and anti-inflammatory agents act as predictors and help in prognosis in ischemic injury. However, other cytokines also contribute to brain damage and repair, but the balance between the beneficial and detrimental effects of cytokines largely depends on the biochemical and physiological status of the brain [158].

### Chemokines

Chemokines are small signaling proteins (8–10 kDa) belonging to the family of cytokines. They have the ability to induce directed chemotaxis in nearby responsive cells, especially leukocytes [159, 160]. There are 40 different chemokines known so far; they all share a similar structural pattern with 4 cysteine residues and, based on that, they are classified into four subfamilies that play a major role in stroke with C-X-C attracting neutrophils and C-C attracting monocytes/macrophages [161]. The other two classes being C and CX3C where Cs denote two N-terminal cysteine residues and depending on whether amino acid is between them or adjacent to them, they are classified as CXC and CC, respectively. Similar to cytokines, chemokines have both unique and overlapping receptors, which belong to the superfamily of G-protein-coupled receptors [162]. Identical to cytokines, chemokines and their receptors are usually expressed in low concentrations [163, 164], but after cerebral ischemia, TNF-α and IL-1β enhance the production and release of specific chemokines such as cytokine-induced neutrophil chemoattractant (CINC), monocyte chemoattractant 1 (MCP-1), microglial response factor-1 (MRF-1), and fractalkine and macrophage inflammatory protein 1 (MIP-1) which are upregulated in the first 3 h and remain elevated for at least 6 h [163]. Chemokines and their receptors play a major role in modulating various pathological and physiological processes, in which their role in post-ischemic inflammation is an important contributor to ischemic brain injury [165]. Brait et al. used PCR arrays to screen temporal expression profile of several chemokine-related genes using focal cerebral ischemia (occlusion for 30 min) in mice. Gene analysis at 4, 24, and 72 h reperfusion showed that several chemokines belonging to CXC family were upregulated (> 10-fold), mediated leukocyte infiltration and played a major role in stroke pathogenesis [166]. Since chemokines have been implicated in the worsening of stroke pathogenesis, their ligands and receptors act as potential therapeutic targets. One such chemokine, chemokine ligand 2 (CCL-2) and its receptor CCR2 signaling, mediates pathological post-ischemic inflammatory response by not only inducing leukocyte recruitment but also disrupts BBB and leukocyte adhesion to brain endothelial cells in an MCAO (45 min occlusion) mice model [167, 168]. CCL-2 levels increase in ischemic penumbra from 6 h of reperfusion with peak levels at 24 and 48 h [169, 170]. Levels of CCL2/CCR2 are positively correlated with infarct size and enlargement of the ischemic lesion. Moreover, CCL2 expression is upregulated in the CSF and serum in ischemic patients [171]. Thus, genetic deletion or manipulation of CCL2/CCR2 expression may be a therapeutic target for ischemic stroke. CCL2 gene disruption diminished infarct volume in focal cerebral ischemic mice model (30 min occlusion), and CCR2 deletion not only reduced infarct size and brain edema but also enhanced motor functions in focal transient cerebral ischemia mice model with 30 min occlusion [169, 172]. Alternatively, overexpression of CCL2 exacerbates ischemic injury in mice [170]. In a recent study using CCR2 knockout mice, after MCAO and reperfusion, the infarct size was less in CCR2 KO mice with lower mortality when compared to WT control when measured 3 days after stroke. Nonetheless, CCR2 KO mice had high mortality and neurological deficit from 5 to 28 days after stroke. Hence, CCR-2-dependent monocyte/macrophages not only aggravate brain injury but also alleviate functional recovery after ischemic stroke. Few reports demonstrated that CCR-2-dependent monocyte infiltration to the stroke-injured hemisphere peaked at 3 days after stroke, but after day 7, monocyte-derived macrophages (MDM) exhibited both proinflammatory and anti-inflammatory phenotype equally, but after 2 weeks, macrophages with anti-inflammatory phenotype dominated. However, blocking monocyte recruitment using anti-CCR2 antibody at 1 week post-stroke eliminates long-term behavioral recovery with significant decrease in anti-inflammatory gene expression in an MCAO mice model with 30 min occlusion [173]. Apart from the anti-inflammatory mechanisms, MDM regulate and control long-term and acute microglia-mediated neuroinflammation [174]. Hence, manipulation of periphery macrophage control of microglia could be a therapeutic option for treatment for microglia-mediated CNS diseases. Apart from proinflammatory properties of CCR2, CCR-2 recruit bone marrow-derived monocytes/macrophages to prevent hemorrhagic infarct transformation and these cells help maintaining neurovascular unit integrity following ischemia in both photothrombotic and tMCAO mice models [175]. Hence, pharmacological manipulation of CCR2 must be deliberately investigated to avoid impairment of normal physiological function of CCR2. Similar to CCL-2, macrophage inflammatory protein 3α (CCL20) has also been suggested to be involved in proinflammatory macrophage recruitment to the ischemic brain followed by IL-1β and TNF-α production [176, 177]. The initial production of chemokines is attributed to activated microglia (MIP-1α, MIP-2, and MRF-1) followed by astrocytes and injured neurons (fractalkine and MCP-1) after cerebral ischemia [72, 144, 178]. Under pathological conditions, such as brain injury, chemokines act as signals released into cerebrospinal fluid (CSF) and extracellular fluid to recruit neutrophils, monocytes, and microglia [179] whereas under normal physiological conditions, chemokines govern the positioning of cells in tissues and recruit leukocytes to the inflammatory site [180]. Leukocyte recruitment is achieved by chemokines working in harmony with adhesion molecules to affect BBB permeability through diapedesis (passage of blood cells through intact walls of capillaries, generally along with inflammation). Kim et al. also proved that mRNA expression of monocyte chemoattractant protein 1 (MCP-1) was nearly undetectable under normal physiological conditions but after ischemia caused either by permanent or temporary MCAO for around 12 h or 2 days, resulted in a significant increase in MCP-1 mRNA expression, which persisted for up to 5 days. Supporting this hypothesis, Chen et al. proved that overexpression of MCP-1 exacerbated ischemic brain injury (24–48 h following occlusion) along with enhanced infiltration of inflammatory cells in MCAO (2 h occlusion) mice model [181]. Similar to MCP-1, hindering the activation of MIP-3-α resulted in reduced infarct size in a transient MCAO rat model with 2 h occlusion [182]. Chemokine overexpression results in chronic neutrophil infiltration, persistent glial activation, and BBB disruption, resulting in terminal wasting syndrome, but on the other hand, chemokine knockout mice show deficiency in leukocyte recruitment [163, 183]. Apart from their chemotactic properties, chemokines directly affect BBB. Co-culture of brain endothelial cells and astrocytes showed that addition of MCP-1 resulted in a significant increase in BBB permeability and it causes alteration in tight junction proteins (TJP) in endothelial cells and the detrimental action of MCP-1 is diminished by the absence of chemokine receptor type 2 [184]. Hence, chemokines may be a potential target for therapeutic interventions.

### Excitotoxicity

Ischemic stroke causes major ATP and phosphocreatinine depletion that results in the release of excitatory amino acids that leads to excitotoxic neuronal damage called excitotoxicity. Barone et al. reported that accumulation of potassium ions and acidosis are preceding events in the ischemic cascade leading to ionic disturbances [185]. Increase in potassium (K^+^) levels leads to the release of glutamate, which in turn stimulates Na^+^/Ca^2+^ channels coupled to *N*-methyl-*D*-aspartate receptors (NMDAR). This further elevates Na^+^ and Cl^-^ levels along with passive influx of H_2_O resulting in cyotoxic edema. Extracellular glutamate also activates α-amino-3-hydroxy-5-methyl-4-isoxazolepropionic acid (AMPA) and metabotropic glutamate receptors, which is a critical step in the inflammatory cascade [186]. Metabotropic and NMDA receptors work through monoionic channels and incidentally enhance intracellular Ca^2+^ levels. Various detrimental pathways including voltage and receptor gated Ca^2+^ influx leads to a significant increase in free cytosolic calcium levels thereby creating mitochondrial calcium overload and further compromising ATP production [185]. Moreover, high intracellular Ca^2+^ levels lead to the activation of proteases, lipases, kinases, phosphatases, endonucleases, and free radicals that promote breakdown of phospholipids, proteins, and nucleic acids [187, 188]. In normal physiological state, Mg ^2+^ blocks channel pores of NMDA receptor, but when glutamate is released from pre-synaptic sites and when AMPA receptors are activated, Mg ^2+^ is completely removed from NMDARs due to partial depolarization in post-synaptic membrane. This causes influx of Na^+^ and CA^+^ into the cell that plays a major role in ischemic cell death [189, 190]. Calcium overload inside neurons activates a series of downstream death signaling pathways such as calpain activation [191], ROS production [192], and mitochondrial impairment [193]. Hence, NMDAR antagonists have been rigorously studied as a therapeutic candidate for treatment of ischemic stroke. In specific, GluN2A- and GluN2B-containing NMDAR are the two important NMDAR in adult forebrain. During an ischemic event, activation of synaptic and extra synaptic GluN2B-containing NMDARs leads to excitotoxicity followed by neuronal apoptosis. Alternatively, activation of GluN2A-containing NMDARs also results in neuroprotection and neuron survival [194, 195]. Hence, dual roles of NMDARs might depend on subcellular locations and subtypes of the receptors that are activated. GluN2B, PSD95, and nNOS complexes play a major role in the activation of death signaling pathways during ischemic stroke [196]. Post-synaptic density protein-95 (PSD-95), a scaffolding protein, links NMDARs to downstream molecules, such as nitric oxide synthase (NOS). PSD-95 is made up of three PDZ domains in which PDZ1 and PDZ2 bind to threnonine/serine-x-valine-COOH (T/SXV) motif at the intracellular c-termini of GluN2 containing NMDAR subunits [197]. Also, PDZ2 of PSD95 binds to N-terminus of nNOS. Both these events lead to Ca^2+^ influx from overactivation of nNOS, followed by excess production of nitric oxide (NO), an effector of excitotoxicity [198]. Hence, disrupting GluN2B-PSD95-nNOS complex impairs NO production and prevents excitotoxicity based neuronal damage [199]. One such study showed that “Tat-NR2B9c or NA-1,” an interfering peptide that binds to either PSD95 or nNOS, prevents the activation of downstream neurotoxic pathways and neuronal superoxide production in neuronal cells [200]. In vitro studies have also demonstrated that administration of Tat-NR2B9c after ischemic stroke reduced infarct volume and improved behavioral outcomes [201]. Cook and his colleagues mimicked clinically relevant situations in a gyrencephalic non-human primate MCAO model and demonstrated that Tat-NR2B9c reduced infarct size (MRI and histology), maintained the ability of ischemic cells to preserve gene-transcription in genome-wide screens, and also prevented behavioral impairment [199]. A double-blinded, randomized, controlled proof-of-concept study conducted across 14 hospitals in the USA showed that NA-1 administration decreased ischemic infarcts. This study was performed on patients who had ruptured or unruptured intracranial aneurysm amenable to endovascular repair because diffusion-weighted MRI showed 90% of patients undergoing endovascular repair show small, embolic procedurally induced ischemic stroke [202]. In addition to peptides, small molecules targeting GluN2B-PSD95-nNOS complex are being studied. In vitro and in vivo studies demonstrated that ZL006 was reported to selectively obstruct PSD95 and nNOS interaction during ischemia. In addition, ZL006 did not affect the normal physiological role of MNDARs and nNOS [196, 203]. Similarly, IC87201 disrupted pathogenic PSD95-nNOS interaction without impairing normal nNOS activity in neurons [204]. However, biochemical and biophysical studies using fluorescence polarization (FP), ^1^H-^15^N.HSQC NMR and isothermal titration calorimetry have shown that under applied in vitro conditions, both ZL006 and IC87201 do not interfere with PDZ domains of nNOS or PDS-95 and it also does not inhibit nNOS-PDZ/PSD-95-PDZ interface [205].

Currently, safety and optimal neuroprotection of Neu2000 in ischemic stroke with endovascular recanalization (SONIC) trial is being performed to evaluate the neuroprotective efficacy of Neu2000 before endovascular thrombectomy in ischemic stroke patients [206]. Neu2000, a sulfasalazine derivative, selectively blocks NMDA receptors along with robust free radical scavenging property. Preclinical animal models demonstrated favorable efficacy and therapeutic window profile [207]. Apart from these small molecules, neuroprotective efficacy of peroxynitrite scavenger, disufenton sodium (NXY-059), uric acid, and antioxidants (edaravone) were evaluated using rodent models and clinical trials (discussed in detail in the “Oxidative stress” section). Free radicals also promote BBB disruption, brain edema and it has been reported that there is a significant decrease in free radical scavenging enzymes (superoxide dismutase) and increase in NO levels during ischemic stroke. In conclusion, decrease or decline in cerebral blood flow drains energy that is required for cellular ionic homeostasis. Ischemia-induced depolarization results in increased glutamate release, which leads to activation of endonucleases [208]. Hence, NMDA and AMPA receptor antagonists can be developed as neuroprotective agents that can inhibit depolarization and prevent ionic perturbations. Glutamate receptor-mediated excitotoxicity is also activated by death molecules like Fas ligand, generated by matrix metalloproteinase, matrilysin [209]. Tissue inhibitor of matrix metalloproteinase 1 (TIMP1) inhibits excitotoxic-mediated neuronal death [210]. Hence, the role of MMPs in aggravating ischemic neuronal death is discussed below.

### Matrix metalloproteinases

Matrix metalloproteinases (MMPs) are a large family of proteolytic enzymes that degrade all components of extracellular matrix [211]. MMPs range from matrilysin (267 amino acids), being the smallest member of the family to large transmembrane proteins such as MMP-14 (582 amino acids). All MMPs have a definitive configuration consisting of the catalytic zinc site, the propeptide region, the fibronectin binding site, and the transmembrane site. MMPs are broadly classified into constitutive (MMP-2 and MMP-14) and inducible (MMP-3 and MMP-9) enzymes, where constitutive enzymes act close to the site of activation whereas inducible enzymes are not constrained to act at the activation site [53]. Although, MMPs act as proinflammatory factor, they are also important for normal physiological function such as neuronal regeneration, cell proliferation, angiogenesis, and apoptosis [212]. MMPs play a major role in BBB disruption during the acute phase of ischemic stroke by degrading basal lamina and weakening the blood vessels. MMP-9, an inducible MMP, is a 92-kDa type IV-collagenase initially secreted in latent form and is activated by proteolytic processing in the extracellular space. Studies using ischemic rodent models demonstrated that there is a significant increase in the expression of pro/active MMP-9 within 24 h following ischemia in rats [213], and they have been detected in both central and peripheral cells with a unique expression profile [214, 215]. MMP-9 along with tissue plasminogen activator (tPA) has been reported to disrupt BBB resulting in hemorrhagic transformation [216]. Rosenberg et al. using an MCAO rat model with 2 h occlusion demonstrated that the activity of MMP-9 was maximally elevated at 48 h [217]. MMP-9 is initially produced by endothelial cells and neutrophils, and after 5 days, they are produced by macrophages. During ischemic stroke, endothelial cells overexpress MMP-9 within and at the periphery of ischemic lesions that results in increased vascular permeability. Type IV-collagen, laminin, and fibronectin are the key components of basal lamina that separates cerebral blood vessels from extracellular matrix. Overexpression of MMP-2 and MMP-9 can digest basal lamina, and this digestion begins as early as 2 h following ischemia, which corresponds with BBB breakdown 3 h following ischemia [218]. Moreover, following stroke, alterations in other MMPs such as enhanced levels of pro/active MMP-2 [219], MMP-3 [84], MMP-10 [219], and MMP-13 [220] have been reported. MMPs have been associated with increase in circulating cytokines [221] and escalation of thrombolysis [222] along with activation of microglia and astrocytes [84]. Various studies demonstrated that MMP inhibition not only reduces infarct size but also alleviates brain edema and hemorrhage [53, 223]. Moreover, when compared to wild-type mice, MMP-9 knockout animals had smaller infarct. Whereas, a similar effect was not observed in MMP-2 knockout mice which demonstrates that MMP-2 may be involved with neovascularization whereas MMP-9 may be involved in edema [224]. Apart from this, MMP levels in plasma could be developed as potential biomarkers since they can be used to predict the severity of stroke. First-ever ischemic stroke patients enrolled in intensive rehabilitation study demonstrated that MMP levels were stable as healthy volunteers during the study period but baseline MMP-12 and MMP-13 were correlated with stroke severity. Surprisingly, plasma MMP3 was significantly increased in patients with better motor/functional recovery [225]. However, serum level of MMP-9 was independently positively correlated with initial stroke severity, as well as with clinical recovery [226]. Since MMPs play both beneficial and detrimental roles in stroke, MMPs are explored as potential therapeutic targets that are reviewed in detail by Yang et al. [53]. Further, MMP-mediated BBB permeability in ischemic stroke is inhibited by cyclooxygenase 2 inhibitors [227]. Hence, modulating the activity of Cox-2 or prostaglandin E2 prevents MMP-mediated BBB disruption.

### Cyclooxygenase—an arachidonic acid metabolite

Activation of immune cells results in the release of phospholipase A2 (PLA2) that leads to initiation of the arachidonic acid (AA) cascade by hydrolyzing glycerophospholipids. This results in subsequent energy failure and depletion of ion concentrations due to intracellular calcium accumulation [228]. Tabuchi et al. demonstrated that AA metabolites act as signaling molecules that initiate a post-ischemic immune response in MCAO (75 min occlusion) mice model. Further, PLA2 deficient mice had small infarcts when compared to wild-type mice demonstrating their detrimental role in brain ischemia [229]. Cyclooxygenase (COX) further metabolizes AA to prostaglandin H2, once released from brain phospholipids. Cox is present in three isoforms namely Cox-1, Cox-2, and Cox-3 [230], where Cox-1 is constitutive and Cox-2 is inducible. Twenty-four and 96 h following brain ischemia by MCA occlusion, Cox-1^−/−^ mice had larger infarcts when compared to COX-1^+/+^ mice which might be due to severe cerebral blood flow reduction in the vulnerable region at the periphery of the ischemic territory [231]. Thus, vascular function of COX-1 plays an important role in maintaining cerebral blood flow in post-ischemic brain. Alternatively, pharmacological inhibition of Cox-1 using Valeryl Salicylate in a model of global cerebral ischemia with 5 min occlusion increased the number of healthy neurons in the hippocampal CA1 even after 7 days post-ischemia [232]. Under normal conditions, Cox-2 is involved in synaptic plasticity and cerebrovascular regulation [233, 234]. Whereas, under disease conditions, their reaction products have a major role in glutamate excitotoxicity [235]. Cox-2, an essential isoform for prostanoid synthesis, has been reported to be enhanced within the ischemic border zone in rat models of focal cerebral ischemia [236]. Autopsy studies have also demonstrated that Cox-2 immunoreactivity has been observed in vascular cells, infiltrating neutrophils and also in neurons sited at the border of an infarct in stroke patients [237, 238]. Prostanoids, a sub-class of eicosanoids, which is reported to be a key factor in the pathological mechanism of ischemic and excitotoxic brain injury, is derived from Cox-2 [235]. Long-term treatment with Cox-2 inhibitors has been shown to elevate the incidence of myocardial infarction and stroke [239]. Hence, it is mandatory to specifically target downstream effectors of Cox-2. Specifically, Cox-2-mediated neurotoxicity is achieved through prostaglandin E2, a downstream effector molecule that acts through four G-protein-coupled receptor namely EP1, EP2, EP3, and EP4 [240, 241]. These receptors have distinct signal transduction profiles and mostly opposite cellular actions [241]. Various studies support the detrimental role of EP1 subtype of prostaglandin E2 receptor in cerebral ischemia [240, 242]. Moreover, administration of EP1 receptor antagonist ONO-8713 followed by striatal unilateral NMDA injection prevents neurotoxicity and diminishes ischemic and excitotoxic brain injury [242, 243]. EP1 receptors augment neurotoxicity by impairing Na^+^–Ca^2+^ exchange leading to disruption of Ca^2+^ homeostasis and resultant excitotoxic neuronal death. Moreover, pharmacological inhibition of EP1 receptor with SC51089 (EP1 antagonist) 6 h after MCA reduced brain injury suggesting their importance for therapeutic development [240].

### Transcriptional modifications

It is well known that following cerebral ischemia, there is upregulation of mitogen-activated protein kinase (MAPK) and nuclear factor kappa beta (NF-κβ) gene expression, which both play a key role in activation of inflammatory signals [244]. NF-κβ family shares a Rel homology, and this heteromeric transcription factor is usually made up of a sequel of Rel subunits such as Rel (cRel), Rel A (p65), Rel B, NF-κβ1 (p50 and its precursor p105), and NF-κβ. The most common composition of NF-κβ is p50 and p65 and is normally found in the cytoplasm bound to its inhibitor protein IκB. IκB kinase (IKK) phosphorylates an inhibitor of kappa B (IκB) that leads to ubiquitination and dissociation of IκB from NF-κβ and eventual degradation of IκB by the proteosome. This process helps NF-κβ to translocate to the nucleus and bind to specific sites of DNA and in promoter domains of proinflammatory genes that lead to transcription of TNF, ICAM-1, COX-2, iNOS, and IL-6. There is a strong correlation between oxidative stress-mediated neurotoxicity and elevated NF-κβ expression. NF-κβ expression contributes to the increase in cell death after MCAO and its activation results in enhanced expression of downstream target genes that play a vital role in neuronal injury. Moreover, either inhibition of p50 or p50 in knockout mice models protects from brain ischemia [245, 246]. Further, Hermann et al. reported that inhibition of IkK markedly reduced infarct size and in contrast activation of IkK enlarged the infarct size. This work is also supplemented by a selective small molecule IkK inhibitor studies that mimicked their genetic studies [247]. Activation of NF-κβ and MAPK pathways also leads to the expression and activation of nucleotide-binding oligomerization domain (NOD)-like receptor (NLR) pyrin domain containing 1 and 3 (NLRP1 and NLRP3) inflammasome protein that contributes to neuronal cell death and brain injury following 1 h occlusion in a MCAO mice model [248].

### Mitogen-activated protein kinase (MAPK)

Mitogen-activated protein kinase (MAPK) family is composed of three groups namely extracellular signal-regulated kinase ½ (ERK ½), c-Jun N-terminal kinases (JNK), and p38 [249]. Various stress factors such as cytokines, osmotic stress, and microtubule disorganization stimulate MAPK pathway that leads to activation of three-tiered Raf/MEK/ERK cascade through G-protein-coupled receptors. Stress activated protein kinases (SAPK), JNK, p38 MAPK, and ERK have been reported to play a detrimental role in brain ischemia [250]. Following brain ischemia, activation of MAPK pathway was noticed to occur 30 min and 3 days, and moreover, many proinflammatory mRNA transcriptions are mediated by p38 MAPK that suggests its role in inflammation-mediated ischemic brain injury. Following ischemia (90 min occlusion), p38 MAPK signaling plays a major role in ischemia-induced astrogliosis, while p38 inhibition attenuated hypoxia and scratch injury-induced astrogliosis in a MCAO mice model [251]. Phosphorylated p38 MAPK was detected in astroglia [252], microglia [253], and neurons [254] of ischemic brain tissue that demonstrates its role in the inflammatory response. Increased inflammatory factors can strongly activate P38 MAPK forming an injury cycle [255]. Following 2-h middle cerebral artery occlusion, MAP Kinase/ERK pathway plays a major role in the expression of MMP leading to BBB breakdown and upregulation of proinflammatory factors [256]. Also, inhibition of the MAPK cascade via suppression of cytokines through anti-inflammatory drugs, which blocks p38 MAPK, arrested the production of TNF-α and IL-1β resulting in neuroprotection [185]. Moreover, treatment with a neuronal membrane lipid precursor (CDP-Choline) after ischemic stroke resulted in the decrease of phosphorylation in ERK 1/2, MEK ½, and ElK-1 transcription factors [19]. Hence, there is increasing evidence that MAPK is a significant regulator of ischemic damage that leads to the possibility of using MAPK as a therapeutic target. Moreover, dioscin, a natural steroid saponin, decreased MAPK phosphorylation and inhibited HMGB1 translocation to the cytosol that resulted in less proinflammatory response [257]. MEK/ERK inhibitor U0126 also decreased HMGB1 expression in reactive astrocytes [258]. These reports suggest that MAPK and HMGB1 pathway act as an important factor in stroke pathology.

### High-mobility group box protein family

Alarmins or danger-associated molecular patterns (DAMPs) are released by dying or necrotic cells, initiating an inflammatory response in ischemic core. DAMPs released in the blood stream also help recruit peripheral immune cells. Several DAMP-s such as nucleic acids, ATP, S100 proteins, and HMGB1 have been found to contribute to the inflammatory response in stroke [259]. High-mobility group box (HMGB) proteins are ubiquitous and abundant DNA binding proteins, and they can act as a trigger of neuroinflammation.A unique structure of HMGB1 helps them to bind to DNA and intracellular proteins to mediate DNA repair and transcription [260, 261]. HMGB1, also known as amphoterin, was initially described as a non-histone DNA binding protein with high electrophoretic mobility. It plays a major role in nucleosomal structure stabilization and binds to the minor groove of linear DNA without sequence specificity resulting in association of nucleoprotein complexes and recruitment of transcription factors. Although HMGB1 plays a major role in the conservation of nuclear homeostasis, it also acts as an extracellular signaling factor involved in cell proliferation, differentiation, and pathogenesis [262]. Various studies reported that HMGB1 is released in the brain after cytokine stimulation and is associated with inflammation [263, 264]. During cellular stress such as stroke, HMGB1 functions as a proinflammatory cytokine [265, 266]. Following cerebral ischemia in mice, HMGB1 translocates from nucleus to the cytoplasm or within 1 h, it disappears from the cells completely [259]. The expression levels of HMGB1 in microglia, astrocyte, and blood vessels increased dramatically 2 h after MCAO in mice [267]. Chin et al. recently demonstrated that HMGB1 protects oligodendrocytes and prevents white matter injury during ischemic stress. Mice injected with glycyrrhizin, a specific inhibitor of HMGB1, resulted in expansion of demyelinating lesion along with exacerbated sensorimotor behavioral deficits [268]. HMGB1 is also released by dying oligodendrocyte to act as an autocrine factor under ischemic condition [269]. Clinically, HMGB1 were found to be significantly higher in serum or plasma of patients with ischemic stroke when compared to age- and sex-matched controls [270]. The expression levels of TLR2 on monocytes either stimulated with or without HMGB1 were evaluated in ischemic stroke patients. Real-time PCR and ELISA assays resulted in higher expression of TLR2 in monocytes of stroke patients stimulated with HMGB1. Anti-TLR2 immunomodulation diminished the expression of IL-17, IL-6, and IL-33 [271]. Faraco et al. reported that HMGB1 promotes induction of iNOS, COX-2, IL-1β, and TNF-α and also increases excitotoxic as well as ischemic neuronal death in vitro [272]. In addition, there is a strong correlation between MMP-9 and HMGB1 levels in ischemic stroke patients, which was associated with poor outcome [273]. Toll-like receptors 2 and 4 and receptor for advanced glycated end products (RAGE) can bind to extracellular HMGB1 and active transcriptional factor NF-κB [274–276]. MyD88, a downstream effector molecule of TLR signaling has been shown to be involved in HMGB1-mediated post-ischemic inflammatory response and enhances stroke lesions when compared to MyD88 knockout mice in fMCAO model [277]. Downregulation of HMGB1 by RNAi (RNA interference) resulted in less microglial activation and reduced infarct volume in rodent MCAO models [278, 279]. Various studies have demonstrated that blocking or modulating HMGB1 by compounds such as statins (atorvastatin, fluvastatin) and by shRNA provided neuroprotective effects against ischemic stroke [280–282].

Hence, similar to other inflammatory mediators, HMGB1 plays an important role in aggravating detrimental events during ischemic stroke. Table 2 summarizes the beneficial and detrimental role of key inflammatory mediators associated with stroke.

**Table 2:** Beneficial and detrimental role of inflammatory factors associated with ischemic stroke

Inflammatory mediators	Produced by	Beneficial	Detrimental	Reference
TNF-α	Macrophages, microglia, neurons	Overexpression of caspases, leukocyte adhesion molecules, and neurotrophic factors enhances endothelial cell dysfunction; modulates extracellular Ca2+ levels and neuronal plasticity; stimulates cerebral microvasculature repair, anti-apoptotic factors, and anti-oxidants; and induce ischemic tolerance	Increases or decreases infarct volume; blocks glutamate uptake, stimulate gliosis and release of neurotoxic mediators; enhance Ca2+ signaling in neurons and apoptosis of endothelial cells, edema formation, BBB disruption, prime endothelium for leukocyte adherence; and upregulate NF-ĸB activation	[65, 283, 284]
IL-6	Macrophages, endothelial cells	Enhances post-stroke angiogenesis associated genes, induction IL-1ra	Endogenous pyrogen, attract T lymphocytes	[285–287]
IL-1α/β	Macrophages, microglia, endothelial cells	Enhances IL-1ra expression and promote survival factors	Increases infarct volume, acts as endogenous pyrogen, promote gliosis, increase neurotoxic mediators, enhance Ca2+ in neurons, induce edema formation and BBB disruption, prime endothelium for leukocyte adherence, upregulate MMP-9	[150, 288, 289]
IL-12	Macrophages, T_H_1 cells	Promote T_H_1 phenotype	Increases infarct volume	[290, 291, 292]
IL-8	Endothelial cells, macrophages	Neutrophil chemoattractant	Increases infarct volume	[65, 293]
MMPs	Microglia, astrocytes, leukocytes	Helps remove extracellular matrix; stimulates plasticity, recovery, and repair Clearance necrotic cell debris	Increases infarct volume, excitotoxicity, BBB disruption; promotes leukocyte adherence and transmigration; increases vasogenic edema and hemorrhagic transformation	[20, 294, 295]
iNOS	Endothelial cells, astrocytes, microglia, leukocytes	Promotes vasodilation, key effector molecule of ischemic preconditioning	Increases infarct volume, Induction of iron loss of cells, Inhibition enzymes DNA replication, stimulates expression of inflammatory mediators	[114, 296, 297]
IFN-γ	NK cells, T cells		Increases infarct volume, enhances inflammatory chemokine interferon inducible protein 10 (IP-10) and T-cell infiltration	[298, 299]
TGF-β	Astrocytes, microglia, macrophages	Reduces infarct volume, gliosis, and brain edema; decreases release ROS and apoptosis; prevent neutrophil adherence; Induces IL-1ra expression and angiogenesis, astrocytic TGF-β limit neuroinflammation	Enhance glial scar formation and β-amyloid precursor	[157, 300]
IL-10	Microglia, macrophages, Treg cells, endothelial cells	Decreases infarct volume, diminishes cytokines release and their receptors expression, prevents astrocytic activation, promotes neuronal and glial survival, reduces leukocyte adhesion		[301, 302]
HMGB-1		Endothelial activation, enhances neuronal survival and neurite outgrowth	Increases infarct volume, vascular permeability, and inflammatory mediators, BBB disruption Activation of microglia, upregulates NF-ĸB expression	[303, 270, 304]
CINC, MIP-1, MCP-1, fractalkine, MRF-1	Microglia, infiltrating immune cells	Promote neuroblast migration, hematogenous cell recruitment, and functional repair; scavenge and repair necrotic tissue and angiogenesis	Enhance leukocyte and neutrophil infiltration, increase BBB disruption and cerebral edema, stimulate phagocytosis and apoptosis, increase cytokine secretion	[305, 306, 307]
ROS	Neurons, microglia, astrocytes, leukocytes		Enhances infarct volume, increased production of ROS, early ROS burst; initiates inflammatory response and lipid peroxidation; disrupts protein biochemistry	[308–310]
NO	Neurons, macrophages, astrocytes, microglia, leukocytes, endothelial cells		Increases infarct volume, induces protein nitrosylation and iron loss of cells, inhibits enzymes for DNA replication, upregulates inflammatory mediators	[296, 114, 311]

**Fig. 3:**
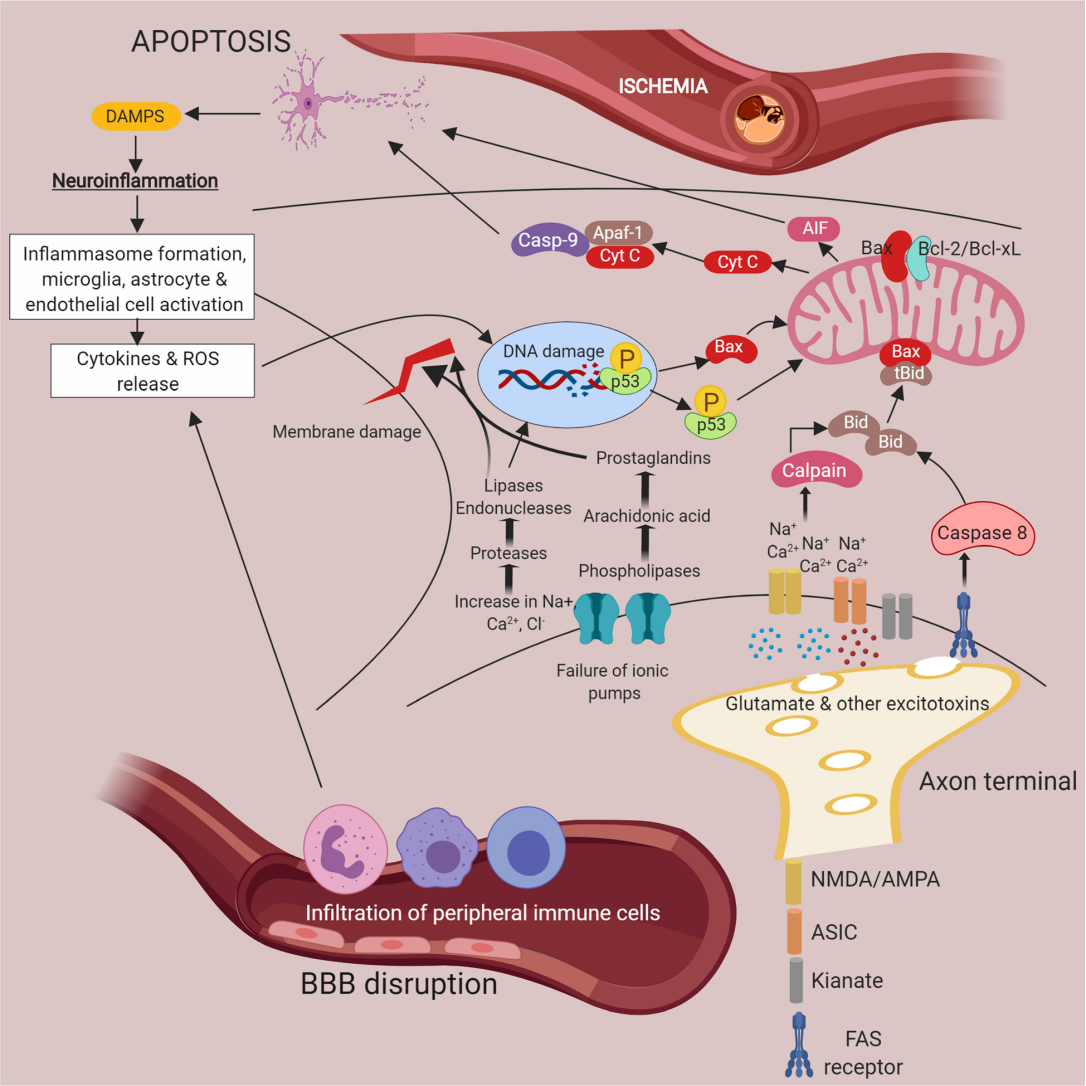
Apoptotic mechanisms involved in ischemic cell death

**Fig. 4:**
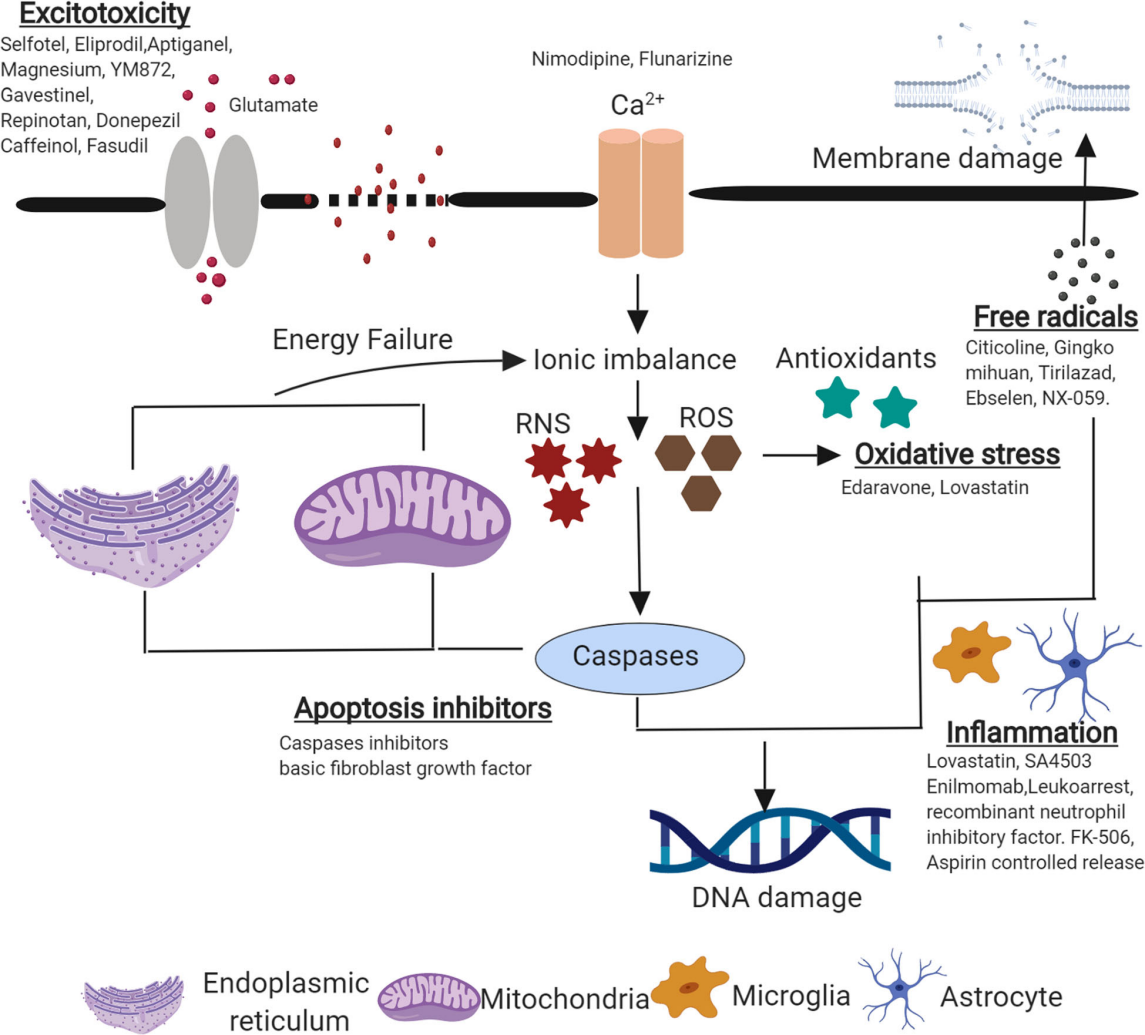
Neuropathological mechanisms in ischemic stroke and respective targets assessed in clinical trials with and without beneficial effects

To conclude, a cascade of these neuroinflammatory events leads to activation apoptosis and resultant cell death. The molecular events initiated in the brain after ischemic stroke involves a cascade of intracellular mechanisms such as failure of ionic pumps, activation of glutamate receptors that leads to excitotoxicity, increase in calcium influx, and enhanced ROS release that leads to DNA damage and mitochondrial impairment. Intracellular calcium influx is enhanced through activation of *N*-methyl-*D*-aspartate (NMDA), D,L-α-amino-3-hydroxy-5-methyl-isoxazolpropionic acid (AMPA) glutamate receptors, or through acid-sensing ion channels (ASICs). Cerebral ischemia leads to increased ROS production and activation of Fas death receptors which results in the activation of pro-apoptotic caspase-8. Increased ROS results in DNA damage and phosphorylation of p53 that activates nuclear cell death pathways. Caspase-8 or calpains activation further leads to cleavage of Bid to truncated Bid (tBid). Truncated Bid fuses with Bax, which in a normal physiological condition, is neutralized by B cell leukemia/lymphoma 2 (Bcl-2) or Bcl-xL. In ischemic state, tBid and Bax interaction leads to mitochondrial membrane depolarization and resultant release of cytochrome C or apoptosis-inducing factor (AIF) into the cytosol. These events initiate caspase-dependent or caspase-independent neuronal death. Released cytochrome C interacts with pro-caspase 9 and apoptotic protein activating factor-1 (Apaf-1) to form apoptosome that leads to the activation of executor caspases such as caspase-3. In addition, AIF transloacates to the nucleus causing DNA fragmentation and resultant cell death. Further, cell death cascade aggravates due to release of damage associated molecular patterns (DAMPs) by damaged neurons that result in activation of microglia, astrocyte, and endothelial cells and inflammasome formation. These events further orchestrate release of cytokines, ROS, and BBB disruption. In addition, BBB disruption causes influx of peripheral immune cells that exacerbates inflammatory pathways (Fig. 3). Numerous studies both in pre-clinical and clinical platforms are being performed in search of novel therapeutic strategies to either inhibit or slow down pathological mechanisms associated with ischemic stroke (Fig. 4). Though various neuroprotective studies have resulted in frustrating clinical trials, their results also provided us with knowledge on understanding the mechanisms involved in ischemic cascades. However, currently, there remains a pressing need for research and development of stroke therapies that can be successfully replicated in clinical trials for prevention as well as for early critical care in stroke patients.

## Conclusion

Accumulating evidence demonstrates that inflammation plays a key role in the pathogenesis of stroke and it has become an interesting target for therapeutic intervention. However, numerous reports indicate that inflammatory cells are involved in a multiphasic role (beneficial and detrimental) where inhibiting the same pathway at the wrong time could exaggerate the pathogenesis. Hence, a better characterization of stroke pathophysiology with time-defined treatment might provide a definitive protective strategy. Moreover, when basic research is performed using stroke models combined with relevant clinical conditions such as type 2 diabetes, prior infection, and atherosclerosis, there might be successful translation from experimental stroke studies to clinical significance that could pave the way for future successful stroke therapies.

## Data Availability

Not applicable

## Abbreviations

AMPA: *α*-Amino-3-hydroxy-5-methyl-4-isoxazolepropionic acid
BBB: Blood-brain barrier
CAM: Cell adhesion molecules
CCr2: Chemokine receptor type 2
CINC: Cytokine-induced neutrophil chemoattractant
COX: Cyclooxygenase
CSF: Cerebrospinal fluid
EC: Endothelial cells
ERK ½: Extracellular signal-regulated kinase ½
GFAP: Glial fibrillary acidic protein
GFP: Green fluorescent protein
GPx: Glutathione peroxidase
HBO: Hyperbaric oxygen
HMGB1: High-mobility group box 1
ICAM 1: Intracellular adhesion molecule 1
IL-1β: Interleukin-1β
IκB: Inhibitor of kappa B
JNK: c-Jun N-terminal kinases
LFA-1: Lymphocyte function-associated antigen 1
MAPK: Mitogen-activated protein kinase
MCAO: Middle cerebral artery occlusion
MCP-1: Monocyte chemoattractant 1
MIP-1: Fractalkine and macrophage inflammatory protein 1
MMPs: Metalloproteinases
MRF-1: Microglial response factor-1
NMDA: *N*-Methyl-*D*-aspartate
NOS: Nitric oxidative species
PLA2: Phospholipase A2
ROS: Reactive oxygen species
SAPK: Stress-activated protein kinases
TGF-β: Transforming growth factor-β
TJP: Tight junction proteins
TLR4: Toll-like receptor 4
TNF-α: Tumor necrosis factor-alpha
tPA: Tissue plasminogen activator
